# Development of measures to evaluate youth advocacy for obesity prevention

**DOI:** 10.1186/s12966-016-0410-x

**Published:** 2016-07-26

**Authors:** Rachel A. Millstein, Susan I. Woodruff, Leslie S. Linton, Christine C. Edwards, James F. Sallis

**Affiliations:** 1 San Diego State University/University of California, San Diego Joint Doctoral Program in Clinical Psychology, San Diego, CA 92103 USA; 2 Present Address: Department of Psychiatry, Massachusetts General Hospital, Boston, MA 02114 USA; 3 San Diego State University, School of Social Work, San Diego, CA 92182 USA; 4 Health Policy Consulting Group, San Diego, CA 92182 USA; 5 Department of Family Medicine and Public Health, University of California, San Diego, CA 92013 USA

**Keywords:** Built environment, Food environment, Physical activity environment, Adolescent, Psychometrics

## Abstract

**Background:**

Youth advocacy has been successfully used in substance use prevention but is a novel strategy in obesity prevention. As a precondition for building an evidence base for youth advocacy for obesity prevention, the present study aimed to develop and evaluate measures of youth advocacy mediator, process, and outcome variables.

**Methods:**

The Youth Engagement and Action for Health (YEAH!) program (San Diego County, CA) engaged youth and adult group leaders in advocacy for school and neighborhood improvements to nutrition and physical activity environments. Based on a model of youth advocacy, scales were developed to assess mediators, intervention processes, and proximal outcomes of youth advocacy for obesity prevention. Youth (baseline *n* = 136) and adult group leaders (baseline *n* = 47) completed surveys before and after advocacy projects. With baseline data, we created youth advocacy and adult leadership subscales using confirmatory factor analysis (CFA) and described their psychometric properties.

**Results:**

Youth came from 21 groups, were ages 9–22, and most were female. Most youth were non-White, and the largest ethnic group was Hispanic/Latino (35.6 %). The proposed factor structure held for most (14/20 youth and 1/2 adult) subscales. Modifications were necessary for 6 of the originally proposed 20 youth and 1 of the 2 adult multi-item subscales, which involved splitting larger subscales into two components and dropping low-performing items.

**Conclusions:**

Internally consistent scales to assess mediators, intervention processes, and proximal outcomes of youth advocacy for obesity prevention were developed. The resulting scales can be used in future studies to evaluate youth advocacy programs.

## Background

Overweight and obesity are global public health, financial, and clinical challenges. The scope of the obesity problem is serious enough that it requires new, larger-scale strategies in addition to those that have already been implemented [1]. Promising solutions for obesity prevention rely on broad-based actions for social, environmental, and political changes that can affect whole populations [2–6].

**Fig. 1:**
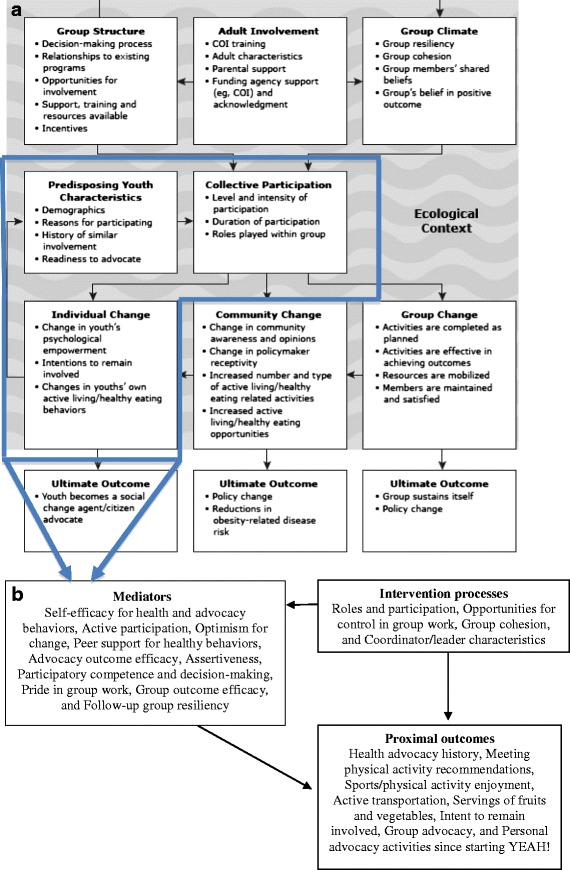
**a** A multi-level conceptual model of processes, evaluation targets, and outcomes of the YEAH! program. Figure reproduced with permission, initially published in [18], adapted from [14]. **b** The parallel constructs and scales developed in the present study. We first published this figure (**a**) in *Preventing Chronic Disease* [18]. We have obtained permission from copyright holders to include the published figure in this article which will be published under Creative Commons (CCBY) license

One promising, though under-studied intervention is advocacy for nutrition and physical activity environment and policy changes. Advocacy refers to the process of increasing support for, recommending, and arguing to promote a cause or policy [7–9]. Youth-oriented groups such as the 4-H Clubs of America (http://www.4-h.org) have a long history of promoting civic engagement and youth empowerment, indicating the benefits of involving youth in the policy process. The American Academy of Pediatrics and Institute of Medicine recognize the need for advocacy and collaboration across sectors to combat obesity [10, 11]. The tobacco control movement’s successes in using youth advocacy to create a social paradigm shift provide a model ready for application to obesity prevention [1, 4, 12, 13]. The American Legacy Foundation’s Statewide Youth Movement Against Tobacco Use (SYMATU) examined the conceptual and practical factors involved in successful youth empowerment and advocacy programs in tobacco control [14]. A related line of research aiming to increase youth physical activity and nutrition showed that improving youth’s proxy efficacy, a construct underlying advocacy, mediated physical activity changes [15–17]. Based on SYMATU and related studies, our group developed a conceptual framework for youth empowerment for obesity prevention that included the following domains: predisposing youth characteristics, collective participation, group structure, adult and institutional involvement, and group climate [18]. Outcomes were conceptualized at the individual, group, community, and society-wide levels [18]. See Fig. 1 for a representation of the proposed mediator, process, and outcome variables in the present study, within the context of the larger evaluation study.

A precondition of developing an evidence base for youth advocacy for obesity prevention is availability of measures. However, no validated evaluation tools designed specifically for youth and their adult leaders could be located at the time the present study began. Thus, several surveys were developed by our group based on our model of youth advocacy for obesity prevention [1] and relevant published measures from other fields, when available. The goal of the present study was to test the psychometric properties of the surveys used to evaluate a youth obesity prevention advocacy program, Youth Engagement and Action for Health (YEAH!), though the measures were designed for wider use. The surveys were tested by creating subscales to measure youth, adult, and group experiences with advocacy, and describing the subscales’ psychometric properties. The hypothesis was that the newly-constructed subscales would demonstrate acceptable internal reliability, fit, and factor loadings in confirmatory factor analysis (CFA). As no published data focused on youth advocacy in the obesity prevention context, analyses were considered exploratory.

## Method

### Procedures

#### Background, recruitment, and inclusion criteria

YEAH! was designed by the San Diego County Childhood Obesity Initiative (SDCCOI) to engage youth and adult group leaders in community advocacy for school and neighborhood improvement projects that impact nutrition and physical activity environments (http://ourcommunityourkids.org/domains--committees/community/youth-engagement--action-for-health.aspx). See [18] for details of the YEAH! program and evaluation study, which additionally included adult group leader and decision-maker interviews. Briefly, the SDCCOI held biannual half-day “train-the-trainer” seminars for adult leaders of youth groups in San Diego County, CA. During these trainings, adults were introduced to the YEAH! manual, which included instructions on implementing community audits of modifiable environment factors, choosing a meaningful project, using assessment tools, developing an advocacy action plan, and advocating for changes. Adult leader participants in the evaluation study were recruited through these trainings.

The main criterion for participating in the present study was membership in an active youth group that focused on advocacy for nutrition or physical activity environment or policy change. Groups could be located or formed in any setting (schools, clubs, religious, military, or other community groups), and they were often in low socioeconomic areas. The youth, leader, and a parent must have provided informed consent (adult leader and parent) or assent (youth). Youth and adult leaders received gift cards and groups received a small stipend as participation incentives. This research project and all procedures were approved by the San Diego State University Institutional Review Board.

#### Intervention and advocacy projects

A brief description is provided; see [18] for details of the YEAH! training and advocacy process. Advocacy projects were designed to be conducted in the following sequence. The adult group leader introduced interested youth to the concept of the built environment’s role in health behaviors, and the group then chose and conducted one (or more) of five environmental audits: school/cafeterias, parks, fast food, stores, or outdoor food advertising. The youth took a checklist and cameras on their selected audit(s) to document potential environmental problems (photovoice). Example targets of change were high prevalence of fast food restaurants around a school, broken or non-existent sidewalks in a neighborhood or around a school, litter/graffiti in local parks, and schools with unhealthy food/beverage vending machines. Once the youth finished their audits, they compiled their findings into an advocacy presentation to be given to a relevant decision-maker(s), e.g., school principal, school nutrition staff, and city council members. The advocacy presentations included the youth’s photovoice documentation of the relevant problems, suggested solutions, and a proposed timeline for requested changes. The YEAH! manual provided recommendations for regular weekly meetings (2–4 h/week) including training, assessment, and advocacy periods extending over several months. Youth and adult leaders were surveyed before and after their advocacy presentations.

### Theory, measures, and instrumentation

Social Cognitive Theory [19] was applied to guide the survey development, given that its emphases on modeling, outcome expectancy, self-, collective-, and proxy-efficacy, and motivation are well-matched with the expected mediators of advocacy behaviors [1]. Survey content was drawn from the guiding conceptual model [1]: education, skill development, behaviors, informed public participation, and engagement. When relevant, we used or adapted items from SYMATU that were based on Empowerment Theory and assessed attitudes and beliefs (e.g., self-efficacy, perceived socio-political control), knowledge and skills (e.g., assertiveness, advocacy experience, decision-making skills, participatory competence, perceived advocacy barriers), collective participation (e.g., reason for joining, level of involvement with other organizations) and group characteristics (e.g., outcome efficacy, group resiliency) [14, 20]. Many factors included in Social Cognitive and Empowerment Theories such as modeling, outcome expectancies, collective efficacy, self-efficacy, participation, and awareness were expected to lead to youth health behavior change. Self- and collective-efficacy and increased engagement and understanding of one’s environment were thought to increase advocacy behaviors.

Basic youth nutrition and physical activity recommendations were included in the YEAH! manual. Given the program’s overarching goal of engagement in obesity prevention, and to be able to assess whether advocacy-related constructs were associated with nutrition and physical activity, these outcomes were included in the present study. We considered behavioral outcomes as potential co-benefits because the curriculum was not designed to promote these as individual behavior changes. We added measures of current levels of physical activity [21], fruit and vegetable, and food and beverage consumption [22] using previously validated measures for adolescents. Additional measures important to obesity were included, such as availability of fast food within a 10-min walk from home or school, food store access, school vending machine access, school lunch options, and outdoor food/beverage advertising. These were drawn from validated instruments [23–28] (for measures and psychometrics, see http://sallis.ucsd.edu/measures.html).

#### Youth baseline survey

The baseline youth survey (paper and pencil) inquired about participants’ current physical activity and nutrition behaviors, attitudes toward advocacy, current advocacy behaviors, and psychosocial variables related to advocacy outcomes (e.g., self-efficacy, leadership confidence, perceived socio-political control). The main aims of the survey were to obtain information about what characteristics are common to participants in these types of groups, as well as mediators (attitudes), intervention processes (group-level factors), and proximal outcomes (advocacy, diet, physical activity behaviors) that might be influenced by participation in advocacy projects. This survey took 15–20 min to complete.

#### Youth follow-up survey

The follow-up youth survey was given to those who completed the baseline survey, at the conclusion of their advocacy projects. This survey had additional scales, including perceptions of group dynamics and leader’s style, their level of group participation, and what they gained from participation. The follow-up survey took no more than 30 min to complete.

#### Adult baseline survey

Adult group leaders were given online surveys (about 20 min each). The baseline survey asked about their leadership experiences; knowledge, attitudes; behaviors surrounding nutrition, physical activity, and advocacy; how many hours per week they expected to devote to this project; and whether they were being paid or volunteering.

#### Adult follow-up survey

The adult group leaders took a longer follow-up survey at the conclusion of their advocacy projects. It asked about any changes in behaviors, attitudes, and knowledge of the aforementioned target outcomes. It inquired about their level of participation in the group decision-making processes, their leadership style, perceptions of group dynamics, problems encountered, and narrative sections to describe what they learned, wished they could do differently, and perceived contributors to success.

### Data analyses

All proposed youth subscales with three or more items were analyzed using CFA with maximum likelihood estimation in MPlus version 6.1 (Muthen & Muthen, Los Angeles, CA) from the baseline survey (*n* = 136). The two-item scales were initially assessed in MPlus, but the CFA results were determined to be unstable given the low sample size. Therefore, for the two-item scales, SPSS version 19 (SPSS Inc., Chicago, IL) was used to conduct principal components analyses, and factor loadings were reported using varimax rotation.

Items were first screened for variability. CFA was used to determine if the a priori factor structure held and to create the subscales for the four surveys. Dimensions (factors) were created in an iterative manner, using fit indices, subscale internal reliability and inter-item correlations, factor loadings (λ), and theory as guides. For the CFA analyses in MPlus, model fit was determined using common recommendations [29] and checked using two types of fit indices. First, a *χ*^2^ test was used to compare the model to the actual data to see if it differed significantly (desired *p*-value > .05). Second, descriptive fit indices were used to evaluate the performance of the factor structure: the comparative fit index (CFI) should be > .93 [30], and root mean squared error of approximation (RMSEA) and standardized root mean residual (SRMR), absolute indexes of overall model fit, should be < .08, with < .06 indicating a better fit for RMSEA [31, 32]. If the model fit based on these statistical criteria, practical significance of the factor loadings was examined using the generally accepted standard of λ ≥ .30 [33], indicating that the factor was at least moderately correlated with the latent variable as proposed. The test of significance for a factor loading indicates that it is significantly different from zero.

Subscale scores from the factors were computed as the mean of the included items, and their internal reliability was checked using Cronbach’s alpha or inter-item correlations (for two-item subscales). Correlations and Cronbach’s alpha values of .70 were considered to be acceptable, but correlations were interpreted with caution due to low sample sizes. If correlations were lower, we examined those values in combination with the other relevant fit indices to determine scale acceptability. Descriptive statistics (means and standard deviations (SDs), frequency distributions) were conducted on all demographics and baseline and post-test subscales to examine distributions of the created subscales. Missing data were not included in these analyses, given a low percentage of missing data in this study.

## Results

Youth baseline demographic and advocacy group characteristics have been published [18]. Briefly, youth came from 21 advocacy groups, age ranged from 9 to 22 years, and were about 2/3 female. Most youth were non-White, and the largest ethnic group was Hispanic/Latino (35.6 %). Most youth’s group advocacy projects focused on schools (67.0 %). Most youth reported previous advocacy experience (72.1 %), and of those who completed the follow-up survey, 60.3 % reported having met with a decision-maker.

### Confirmatory factor analyses of proposed advocacy subscales among youth and adults

Six of the 20 originally proposed youth subscales required modifications, while 14 demonstrated acceptable fit and were unmodified. Table 1 presents CFA and inter-item correlation results for each subscale, items included or dropped based on CFAs, and resulting modifications. Final scale fit indices for the multi-item subscales are presented in the Appendix. Some items were asked as checklists and were not factor analyzed but subscales were created (Reasons for joining, Level/history of prior involvement, Group advocacy, Roles and participation, Benefits of participating). The single-item scales were Knowledge of resources, Social support for health behaviors, Opportunities for involvement in group, and Collective efficacy toward group goals. Table 1 is structured in the following order: hypothesized mediators, intervention processes, and proximal outcomes of youth advocacy.

**Table 1:** Confirmatory Factor Analysis (CFA) Results and Inter-item Correlations of Youth and Adult Subscales with Two or More Items

Subscale	# items in final scale	Items (baseline wording)	Inter-item correlations	Factor loadings (rotated, or unrotated if only 1 factor)
Youth subscales:				
Mediators matched pre- and post- test				
Self-efficacy for health and advocacy behaviors	3	- I am sure that I can tell my friends to eat healthy. - I am sure that I can tell my friends to be physically active. - I am confident that I can work to make my school or community a better place for being physically active and eating healthy.	1.0, .704, .704 α = .68	.840 .801 .390
Perceived sociopolitical control (resulted in two factors)				
Active participation	2	- I like to wait and see if someone else is going to solve a problem. (reverse coded) - I find it very hard to talk in front of a group. (reverse coded)	1.0	.787 .755
Optimism for change	2	- If I tell someone “in charge”, like a leader, about my opinions, they will listen to me. - I enjoy participation because I want to have as much say as possible in my school or community.	.311	.834 .763
Peer support for healthy behaviors (after revision)	2	- How many of your five closest friends are physically active at least 5 days a week? - How many of your five closest friends eat at least 5 servings of fruits and vegetables a day?	.491	.820 .822
Advocacy outcome efficacy	2	- This project can make a difference in making our school or community a better place for being physically active and eating healthy. - This group can influence how people feel about nutrition or physical activity.	.765	.828 .828
Assertiveness (after revision)	3	- I can talk with adults about issues I believe in. - I can ask others to help work on making our school or community healthier. - I can start discussions with others about how to change our school or community to make it healthier.	.474, .524, .678 α = .79	.867 .770 .601
Participatory competence and decision-making	2	- If I have a problem when working towards a goal, I usually do not give up. - I can influence the decisions my group makes.	.268	.796 .796
Post-test only				
Pride in group work	2	- I am proud of the work our group did. - Our work was worth the time and effort we put into it.	.818	.953 .953
Group outcome efficacy	2	- This group can influence how adults in the community feel about nutrition and physical activity. - This group can influence how people my age, who are not in this group, feel about nutrition and physical activity.	.638	.905 .905
Follow-up group resiliency	2	- This group does not give up during tough times. - If this group failed to accomplish one of our goals, we kept trying to find a way to reach it.	.317	.811 .811
Intervention processes post-test only				
Roles and participation: Likert	2	- When I attended meetings, I took part in the discussions. - I took responsibility for things that the group needs to have done.	.389	.836 .836
Opportunities for control in group work	2	- This group allowed me to have a say in planning events or activities. - This group had specific leadership roles for youth.	.481	.860 .860
Group cohesion (after revision)	2	- Members of our group do not spend time together outside of meetings or events. (reverse coded) - I’m unhappy with my group’s level of commitment to its goals for creating healthier communities. (reverse coded)	.202	.775 .775
Coordinator/leader characteristics	3	- Our leader(s) provided help whenever we needed it. - Our leader(s) did not force his or her ideas and opinions on the group. - Our leader(s) let us work through our disagreements to decide what was best for the group.	.253, .317, .424 α = .56	.703 .789 .819
Proximal outcomes matched pre- and post- test				
Health advocacy history	2	- In the last year, how many times have you tried to tell other students, your family, or friends to think more about eating healthy or being physically active - In the last year, how many times have you tried to tell school leaders, people in your community, or politicians to be more interested in making your school or community a better place for being physically active and eating healthy.	.335	.817 .817
Meeting physical activity recommendations	2	- Over the past seven days, how many days were you physically active for at least 60 min per day? - Over a typical week, on how many days are you physically active for at least 60 min per day?	.717	.927 .927
Sports and active transport (resulted in two factors)				
Sports/Enjoyment of physical activity	2	- Not counting PE classes, how many days per week do you play or practice a team sport, or take a physical activity class? - I enjoy physical activity.	.036	.669 .739
Active transport	2	- In a typical week, how many days do you walk or bike TO school? - In a typical week, how many days do you walk or bike FROM school?	.765	.938 .940
Servings of fruits and vegetables	2	- In a typical day, how many servings of fruit do you eat? - In a typical day, how many servings of vegetables do you eat?	.434	.847 .847
Intent to remain involved	2	- I plan to continue to work for change in my school or community after this project is over. - If I had a chance to join a similar group in the future, I would do it.	.562	.884 .884
Post-test only				
Group advocacy (Only if group met with a decision-maker; *n* = 86)				
Group advocacy (after revision)	6	- The decision-maker(s) listened carefully to our group. - The decision-maker(s) seemed to understand what we were asking for. - The decision-maker(s) seemed to learn something new from what we were saying. - The decision-maker(s) would have listened to us more if we were adults instead of youth. - The decision-maker(s) were impressed by our group’s work. - The decision-maker(s) are going to make some changes based on the information from our group.	.424 to .838 α = .47	.157 to .717
Personal advocacy activities since starting YEAH!	2	- Since I started this project, I have talked to my parents or family members about changes needed to make my school or community a better place for being physically active and eating healthy. - Since I started this project, I have talked to my friends about changes needed to make my school or community a better place for being physically active and eating healthy.	.620	.920 .920
Adult post-test				
Group efficacy (leader perspective)	8	How would you rate the success…on the youth? - Building leadership skills - Increasing their knowledge of physical activity and healthy environments - Increasing their knowledge of healthy eating - Increasing their knowledge about the role of policy and environment in supporting healthy eating and physical activity - Building advocacy skills among the youth - Engaging the youth in their communities/neighborhoods - Building self-efficacy among the youth - Educating decision-makers	.099 to .700 α = .82	.431 to .872
Group cohesion and participation (after revision)	3	- Attendance by group members was consistent and strong. - All group members participated enthusiastically. - A few leaders emerged among youth members. - Decision-making was primarily driven by adult leaders. - The youth in the group did not know each other before joining the group.	.295, .348, .701 α = .72	.619 .787 .892

*Note*: Strikethrough items are those that were dropped during factor analysis

### A. Subscales assessing mediators of youth advocacy

Matched pre- and post-test youth mediator subscales were Self-efficacy for health and advocacy behaviors, Perceived sociopolitical control (Active participation and Optimism for change), Peer support for healthy behaviors, Advocacy outcome efficacy, Assertiveness, and Participatory competence and decision-making. Mediator subscales assessed at post-test only were Pride in group work, Group outcome efficacy*,* and Follow-up group resiliency. Six of the initially proposed nine mediator subscales performed acceptably, based on statistical and descriptive fit indices, and were retained as proposed: Self-efficacy for health and advocacy behaviors, Advocacy outcome efficacy, Participatory competence and decision-making, Pride in group work, Group outcome efficacy*,* and Follow-up group resiliency.

Three mediator subscales did not fit well statistically or descriptively and required modifications. The latent Perceived sociopolitical control variable was initially indicated by four items. This one-factor model did not fit well statistically (*χ*^2^ [3, *N* = 136] = 100.36, *p* < .001) or descriptively (CFI = 1.0, RMSEA < .01, SRMR < .01). The standardized factor loadings were low and not statistically significant (.090, .227, –.289, -1.08). Given the poor fit, modification indices that change the model assumptions were added, but the suggested changes that resulted did not significantly improve fit. This proposed factor was split into two two-item subscales based on factor loadings: Active participation and Optimism for change, which were then assessed in SPSS and performed acceptably (Table 1).

The Peer support for healthy behaviors latent variable was first indicated by three items. This one-factor model did not fit well statistically (*χ*^2^ [3, *N* = 136] = 43.28, *p* < .001), but did fit well descriptively (CFI = .97, RMSEA < .01, SRMR < .01). Two standardized factor loadings were large but one was small and all were statistically significant (.270, .683, .731). The “readiness” item was dropped due to a factor loading < .30 and the model was re-run. The resulting two-item one-factor model had large factor loadings (Table 1).

The Assertiveness latent variable was indicated by four items. This one-factor model fit well statistically (*χ*^2^ [2, *N* = 136] = 2.66, *p* = .26) and descriptively (CFI = .99, RMSEA = .05, SRMR = .02). The standardized factor loadings for three of the items were large and statistically significant (.889, .764, .589). The “I am a leader” item did not load highly enough (λ = .203), however, it was statistically significant (*p* < .05). The CFA model was re-run without the leadership item. This three item model did not fit well statistically (*χ*^2^ [3, *N* = 136] = 129.06, *p* < .001, but did fit well descriptively (CFI = 1.0, RMSEA < .01, SRMR < .01). The standardized factor loadings for the three items remained large and statistically significant (λs = .867, .770, .601; Table 1). The three-item factor was determined a better fit and retained.

### B. Subscales assessing intervention processes measured at post-test only

Three of these intervention process subscales fit acceptably and were retained as originally proposed: Roles and participation (Likert scale), Opportunities for control in group work*n* and Coordinator/leader characteristics (Table 1).

The Group cohesion subscale required modification. A one-factor model was initially indicated by three items, and the loadings/principal component extractions were moderate to large (.597, 611, .765). The inter-item correlations were .062, .195, and .202. The item with the lowest correlation and loading was dropped, forming a two-item scale. This two-item model demonstrated a large loading and was subsequently used (Table 1).

### C. Subscales assessing proximal outcomes of youth advocacy and behavior change

There were four matched pre- and post-test proximal outcome subscales and two subscales assessed at post-test only. Five of the initially proposed seven proximal outcome subscales performed acceptably and were retained as proposed: Health advocacy history, Meeting physical activity recommendations, Servings of fruits and vegetables, Intent to remain involved, and Personal advocacy activities since starting YEAH! The physical activity and nutrition scales have previously demonstrated reliability and validity [21, 22].

Two mediator subscales did not fit well statistically or descriptively and required modifications. The latent Sports and active transportation measure was indicated by four items. Lack of descriptive or statistical fit resulted in the proposed factor being split into two two-item subscales, based on factor loadings: Sports/physical activity enjoyment and Active transportation, which both had acceptable factor loadings and inter-item correlations (Table 1).

A one-factor Group advocacy model was initially indicated by seven items. The one-factor model was tested for fit, but two factors emerged. The inter-item correlations ranged from -.086 to .509. Six items loaded on one factor, with moderate to large loading values (λs = .421 to .836). One item (“The decision-makers listened to us more because we were youth […]”) loaded on a second factor (λ = .820). This was the only negatively correlated item so it was dropped, resulting in a six-item subscale. The six-item one-factor Group advocacy subscale was tested for fit. The loading/principal component extractions were mostly large, with one item loading moderately (Table 1).

### D. Adult post-test subscales

Most of the adult group leader items were qualitative or single-item responses and did not form calculable subscales. Adult group leaders and their groups’ characteristics have been previously published [18].

The confirmatory factor analyses of the two multi-item scales are as follows, both from the follow-up survey (Table 1).

Group efficacy (leader perspective). A one-factor Group efficacy model was initially indicated by eight items. This model fit well statistically and descriptively, and standardized factor loadings were high or moderate and statistically significant (Table 1).

A one-factor Group cohesion and participation model was indicated by five items. This one-factor model fit well statistically (*χ*^2^ [5, *N* = 43] = 5.65, *p* = .34) and descriptively on two of three indices (CFI = .98, RMSEA = .05, SRMR = .09). Two standardized factor loadings were high or acceptable (λs = .310, .673) but three loadings were low (λs = .-.041 to .134). No factor loadings were statistically significant. Models with additional paths suggested by modification indices were conducted in MPlus, but none of them improved model fit. The five-item one-factor model was tested for fit and two factors emerged; inter-item correlations: -.128 to .701. Three items loaded on one factor, Group cohesion and participation: strong attendance, group members enthusiastically participated, and a few youth leaders emerged (Table 1). Two items (adult-driven decision-making and the youth did not know each other previously-reverse coded) loaded on a second factor (λs = .879, .501). However these items were virtually un-correlated (.053), so forming a scale was unjustifiable. The final scale consisted of the three items listed above.

#### Youth subscale characteristics

Table 2 presents the descriptive characteristics of these youth and adult CFA-based subscales, along with the subscales based on single-items and checklists. The subscales showed a range of response scales, agreement, and experiences. Of the self-rated youth scales on a 5-point response scale, active participation had the lowest mean (2.62, SD = .96) and group resilience had the highest mean (4.46, SD = .74). There were generally high ratings on the subscales that assessed participants’ evaluation of their groups, indicating positive experiences among those who finished the YEAH! program.

**Table 2:** Confirmatory Factor Analysis (CFA)-Derived Subscales, Single Items, and Checklist Descriptive Statistics: Youth Baseline (*n* = 131–136) and Adult Follow-up (*n* = 45) Sample

Subscale	# items	Mean (SD)	Range
*Youth subscales*			
*Mediators*			
Self-efficacy for health and advocacy behaviors	3	3.82 (.84)	1.33–5
Perceived sociopolitical control			
Active participation	2	2.62 (.96)	1–5
Optimism for change	2	4.04 (.73)	1–5
Peer support for healthy behaviors	2	2.67 (1.20)	0–5
Advocacy outcome efficacy	2	4.36 (.63)	2–5
Assertiveness	3	3.72 (.91)	1–5
Participatory competence and decision-making	2	3.94 (.68)	2–5
Pride in group work	2	4.66 (.61)	1.5–5
Group outcome efficacy	2	4.22 (.77)	2.5–5
Follow-up group resiliency	2	4.27 (.72)	3–5
Knowledge of resources	1	3.48 (1.15)	1–5
Social support for health behaviors	1	3.45 (.81)	1–5
Intervention processes			
Roles and participation: Likert	2	4.22 (.67)	2–5
Roles and participation: checklist	8	1.73 (1.26)	0–5
Opportunities for control in group work	2	4.00 (.87)	1.5–5
Group cohesion	2	3.98 (.84)	1.5–5
Coordinator characteristics	3	4.42 (.67)	2.67–5
Benefits of participating (checklist)	10	6.28 (2.07)	0–10
Opportunities for involvement in group	1	4.19 (1.01)	1–5
Collective efficacy toward group goals	1	4.56 (.71)	2–5
Group resiliency	1	4.46 (.74)	2–5
Proximal outcomes			
Health advocacy history	2	1.81 (1.02)	0–4
Meeting physical activity recommendations	2	3.71 (1.91)	0–7
Sports and active transportation (split into two subscales):			
Sports/Enjoyment of physical activity	2	3.04 (1.20)	.5–5
Active transport	2	1.07 (1.79)	0–5
Servings of fruits and vegetables	2	2.17 (1.02)	0–4
Intent to remain involved	2	4.03 (.81)	2–5
Group advocacy	6	4.26 (.56)	2.67–5
Personal advocacy activities since starting YEAH!	2	3.77 (1.00)	1.5–5
Fast food times per week (*n* = 125)	1	1.69 (1.94)	0–14
Fast food times per month (*n* = 125)	1	5.90 (6.36)	0–30
Level/history of prior involvement (checklist; sum of responses)	8	1.05 (1.17)	0–4
Adult group leader subscales			
Group efficacy	8	3.98 (.56)	2.13–5
Group cohesion and participation	3	3.89(.81)	1–5

## Discussion

There were varying degrees of support for the quality of the CFAs of youth advocacy subscales. The proposed factor structure held for 14 of the 20 originally proposed multi-item youth subscales. The modifications for six scales involved splitting a larger subscale into two components or dropping low-performing items to improve model fit to an acceptable level. For some of the subscales, items were retained despite lower correlations; this tended to happen more with the two-item scales. Retaining items even with a low inter-item correlation is justifiable because the items and scales were built based on theory [34], and dropping some items would have reduced content validity. Correlations and their significance can be influenced by sample size [35], and the present sample size was modest. Further, we only kept questionable items when their factor loadings were sufficiently high (λ ≥ .30). Given the exploratory nature of this study, the specialized nature of the young advocate sample, and the measures’ grounding in theory, the derived scales can be used for research and evaluation purposes, with some caution and need for replication to determine whether further development is warranted.

The surveys’ designs were based as much as possible on similar constructs from the tobacco youth advocacy literature [14, 20, 36], and youth advocacy for obesity prevention models [1, 18], supporting both content validity and ability to compare results across health behaviors. However, we had a small sample size, and this work can be considered exploratory. In particular, it is not certain that the results of the CFAs are robust. To improve the factor analysis interpretation, we would have benefitted from a larger youth sample size. With approximately 10 to 15 youth per baseline item, we would have the suggested sample size to support robust and well-powered CFA results [34]. Future studies should increase sample size and retention based on lessons learned from the larger evaluation study, including sufficient leader training and support and longer project timeframes [18].

### Strengths and limitations

The present study represented the first theory-driven, systematic study of the measurement of hypothesized mediators, processes, and outcomes related to youth advocacy for obesity prevention. It was an important empirical step forward in the field of youth advocacy for obesity prevention for several reasons. First, content validity of the scales was strengthened by the use of general theories, a youth advocacy-specific model, and prior validated surveys from the tobacco control field. Second, we presented systematic subscale development methods and psychometric results. It will be useful for future evaluation studies to have useable, statistically- and theoretically-driven subscales and surveys for youth and adults. The literature will also benefit from having a consistent set of measures with which to compare studies of youth advocacy interventions.

This study’s power was limited by sample size and should be considered exploratory. As multivariate models require more subjects than were available, the models presented here should be interpreted with caution and replicated in future studies. Scale quality is usually enhanced by more items, but the large number of relevant constructs required short scales to reduce participant burden. Thus, scale psychometrics had to be reduced to some extent to achieve study feasibility.

The use of quantitative surveys is only one method to evaluate advocacy’s processes, mediators, and outcomes. Evaluations of youth advocacy could benefit from multiple approaches, including quantitative surveys, qualitative methods, and network analyses among youth participants. The larger program evaluation of YEAH! included key informant interviews and adult group leader semi-structured interviews, as reported previously [18]. The present measures are designed to serve as one tool in a suite of advocacy evaluation methods.

## Conclusions

Based on recommendations, anecdotes, and internet search popularity, there is a great deal of current policy and practice interest in the potential for youth advocacy for obesity prevention. The present study provides measures that can be used to build evidence regarding the processes and outcomes of youth advocacy for obesity prevention. The measures presented here can be used in other studies, but they should be further validated in larger samples, in different populations, and with different programs. Having a unified, validated set of measures used by forthcoming advocacy studies will allow this field of research to move forward efficiently and methodically. Of note, there are many existing measures of relevant constructs including positive youth development and civic engagement that could be used as complementary measures (https://cyfernetsearch.org/home). There are many implications for policy, practice, and future research, and the present findings underscore the need to expand, modify, streamline, and measure the advocacy process to harness the power and potential of youth advocacy for nutrition and physical activity environment and policy change. It is time to improve the use of evidence by decision-makers, which can be accomplished by improved policy research, inter-sector communication and translation, collaborative media use, and citizen participation [37–40].

## Abbreviations

CFA, confirmatory factor analysis; CFI, comparative fit index; RMSEA, root mean squared error of approximation; SD, standard deviation; SDCCOI, San Diego County Childhood Obesity Initiative; SRMR, standardized root mean residual; SYMATU, Statewide Youth Movement Against Tobacco Use; YEAH!, Youth Engagement and Action for Health!

## References

[1] Millstein RA, Sallis JF (2011). Youth advocacy for obesity prevention: the next wave of social change for health. Transl Behav Med.

[2] Institute of Medicine (2012). Accelerating progress in obesity prevention: solving the weight of the nation.

[3] Institute of Medicine (2005). Preventing childhood obesity: health in the balance.

[4] Mello MM, Studdert DM, Brennan TA (2006). Obesity: the new frontier of public health law. N Engl J Med.

[5] Sallis JF, Glanz K (2009). Physical activity and food environments: solutions to the obesity epidemic. Milbank Q.

[6] Sallis JF, Cervero RB, Ascher W, Henderson KA, Kraft MK, Kerr J (2006). An ecological approach to creating active living communities. Ann Rev Pub Health.

[7] Carlisle S (2000). Health promotion, advocacy and health inequalities: a conceptual framework. Health Promot Int.

[8] Martin J (2010). The role of advocacy. Preventing childhood obesity: evidence policy and practice.

[9] World Health Organization (1995). Advocacy strategies for health and development: development communication in action.

[10] Davis MM, Gance-Cleveland B, Hassink S, Johnson R, Paradis G, Resnicow K (2007). Recommendations for prevention of childhood obesity. Pediatrics.

[11] Sanchez E, Burns AC, Parker L (2009). Local government actions to prevent childhood obesity.

[12] Brownell KD, Warner KE (2009). The perils of ignoring history: Big Tobacco played dirty and millions died. How similar is Big Food?. Milbank Q.

[13] Klein JD, Dietz W (2010). Childhood obesity: the new tobacco. Health Aff.

[14] Holden DJ, Messeri P, Evans WD, Crankshaw E, Ben-Davies M (2004). Conceptualizing youth empowerment within tobacco control. Health Educ Behav.

[15] Dzewaltowski DA, Estabrooks PA, Welk G, Hill J, Milliken G, Karteroliotis K, Johnston JA (2009). Healthy youth places: a randomized controlled trial to determine the effectiveness of facilitating adult and youth leaders to promote physical activity and fruit and vegetable consumption in middle schools. Health Educ Behav.

[16] Dzewaltowski DA, Karteroliotis K, Welk G, Johnston JA, Nyaronga D, Estabrooks PA (2007). Measurement of self-efficacy and proxy efficacy for middle school youth physical activity. J Sport Exerc Psych.

[17] Ryan GJ, Dzewaltowski DA (2002). Comparing the relationships between different types of self-efficacy and physical activity in youth. Health Educ Behav.

[18] Linton LS, Edwards CC, Woodruff SI, Millstein RA, Moder C (2014). Youth advocacy as a tool for environmental and policy changes that support physical activity and nutrition: an evaluation study in San Diego County. Prev Chronic Dis.

[19] Bandura A (1977). Self-efficacy: toward a unifying theory of behavioral change. Psychol Rev.

[20] Holden DJ, Evans WD, Hinnant LW, Messeri P (2005). Modeling psychological empowerment among youth involved in local tobacco control efforts. Health Educ Behav.

[21] Prochaska JJ, Sallis JF, Long B (2001). A physical activity screening measure for use with adolescents in primary care. Arch Ped Adol Med.

[22] Prochaska JJ, Sallis JF (2004). Reliability and validity of a fruit and vegetable screening measure for adolescents. J Adol Health.

[23] McKenzie TL, Marshall SJ, Sallis JF, Conway TL (2000). Student activity levels, lesson context, and teacher behavior during middle school physical education. Res Q Exerc Sport.

[24] McKenzie TL, Sallis JF, Prochaska JJ, Conway TL, Marshall SJ, Rosengard P (2004). Evaluation of a two-year middle-school physical education intervention: M-SPAN. Med Sci Sports Exerc.

[25] Patrick K, Calfas KJ, Norman GJ, Zabinski MF, Sallis JF, Rupp J, Covin J, Cella J (2006). Randomized controlled trial of a primary care and home-based intervention for physical activity and nutrition behaviors: PACE+ for adolescents. Arch Ped Adol Med.

[26] Patrick K, Norman GJ, Calfas KJ, Sallis JF, Zabinski MF, Rupp J, Cella J (2004). Diet, physical activity, and sedentary behaviors as risk factors for overweight in adolescence. Arch Ped Adol Med.

[27] Grow H, Saelens B, Kerr J, Durant N, Norman G, Sallis JF (2008). Where are youth active? roles of proximity, active transport, and built environment. Med Sci Sports Exerc.

[28] Forman H, Kerr J, Norman GJ, Saelens BE, Durant NH, Harris SK, Sallis JF (2008). Reliability and validity of destination-specific barriers to walking and cycling for youth. Prev Med.

[29] Bentler PM (2007). On tests and indices for evaluating structural models. Pers Indiv Dif.

[30] Bentler PM (1990). Comparative fit indexes in structural models. Psychol Bull.

[31] Hu LT, Bentler PM (1999). Cutoff criteria for fit indexes in covariance structure analysis: conventional criteria versus new alternatives. Struct Equ Modeling.

[32] Steiger JH (1990). Structural model evaluation and modification: an interval estimation approach. Multivariate Behav Res.

[33] Tabachnick BG, Fidell LS (2007). Using multivariate statistics.

[34] Meyers LS, Gamst G, Guarino A (2006). Applied multivariate research: design and interpretation.

[35] Bates BT, Zhang S, Dufek JS, Chen FC (1996). The effects of sample size and variability on the correlation coefficient. Med Sci Sports Exerc.

[36] Winkleby MA, Feighery EC, Altman DA, Kole S, Tencati E (2001). Engaging ethnically diverse teens in a substance use prevention advocacy program. Am J Health Promot.

[37] Brownson RC, Royer C, Ewing R, McBride TD (2006). Researchers and policymakers: travelers in parallel universes. Am J Prev Med.

[38] Glasgow RE, Lichtenstein E, Marcus AC (2003). Why don’t we see more translation of health promotion research to practice? Rethinking the efficacy-to-effectiveness transition. Am J Public Health.

[39] Lawrence M, Swinburn B (2010). The role of policy in preventing childhood obesity. Preventing childhood obesity: Evidence, policy, and practice.

[40] McGinnis JM, Williams-Russo P, Knickman JR (2002). The case for more active policy attention to health promotion. Health Aff.

